# Correlate light–matter interactions in different spectral regimes

**DOI:** 10.1038/s41377-022-00724-9

**Published:** 2022-03-03

**Authors:** Qiaoqiang Gan

**Affiliations:** 1 Material Science Engineering, Physical Science Engineering, King Abdullah University of Science and Technology, Thuwal, Saudi Arabia; 2 Electrical Engineering, University at Buffalo, The State University of New York, Buffalo, NY USA

**Keywords:** Optical sensors, Nanophotonics and plasmonics

## Abstract

Using mid-infrared plasmons to trigger visible surface enhanced Raman spectroscopy signals within a nanocavity represents new opportunities for fundamental investigation of light–matter interaction within quantum regimes, requiring improved sensing capabilities enabled by well-designed nano/microstructures and characterization systems.

The unprecedented ability of light concentration within metallic nanostructures has attracted significant research interests in recent years^1^. Using a variety of nanoantenna structures, the incident optical field can be concentrated into deep subwavelength volumes and realize significant localized-field enhancement (so called “hot spots”)^2^, which is promising to develop enhanced nonlinear optics^3^, surface photoctalysis^4,5^ and surface enhanced spectroscopic sensing technologies^6,7^. It is generally believed that smaller gaps between metallic nanopatterns will result in stronger localized field enhancment due to optically driven free electrons coupled within the gap. However, due to the diffraction limit of classic optics, it is a grand challenge to couple and confine the light into these deep subwavelength volumes. Therefore, the capability to manipulate light within these extremely deep subwavelength scales is promising to introduce revolutionary advances in light–matter interaction and pave the way towards light manipulation within extremely small and even quantum regimes^8^.

In a recent issue published in 2021, Wang and Tian et al. overviewed the latest research progress from nano/micro-photonic structures to macro-optical deisngs, and explained their implimentations in surface enhanced Raman spectroscopy (SERS) and infrared absorption spectrocopy technologies^9^. Absorption peaks in infrared absorption spectroscopy generally correspond to Raman peaks in SERS spectra. These two technologies are usually considered as complementary technologies that can produce more sensitive capabilities when performed simultaneously. However, since the vibrational absorption signal of surface enhanced infrared absorption spectroscopy is proportional to |E/E_0_|^2^ in contrast to |E/E_0_|^4^ in SERS, light trapping and localization structures for both infrared wavelengths and visible to near infrared wavelengths are highly desired^10–12^. In addition to the development of better light localization nano/micro-structures, another key insight is that it is essential to combine engineered nano/micro-structures with optimized macro-optical systems to boost the overall sensing capability. In particular, a well-designed system may introduce new capabilities to further manipulate the optical field within the tiny volume, which was not possible before. For instance, an earlier example employed two atomic-force microscope tips with metal particles to manipulate the tip-tip distance accurately^13^. By characterizing the optical scattering spectrum and current simultaneously, a quantum scale limit of 0.3 nm was revealed at which tunneling plasmonics start to dominate, representing the upper limit for localized plasmonic enhancement. With smaller gaps, this enhancement will decrease due to the emergence of quantum-tunneling charge-transfer plasmons, uncovering new areas for quantum plasmonics^8,14^ and extreme nonlinear interactions^3^ within nanometer scales.

Recentlly, Chikkaraddy and Baumberg reported another example to demonstrate this type of combination: i.e. using mid-infrared (mid-IR) plasmons to trigger visible SERS signals within a nanocavity^15^. The authors employed a carefully designed optical system to focus the visible and mid-IR beams to excite plasmons within a designed nanogap and correlated the light–matter interaction in different spectral regimes (i.e., visible and mid-IR). As illustrated in Fig. 1, the nanocavity is constructed by a metallic nano-sphere on a planar metal film separated by a monolayer molecule. This type of cavity has been explored expensively in recent years to boost the light–matter interaction and even approach the quantum upper limit of the localized field enhancement at the contact point between the sphere and the planar film (e.g., ref. ^14^). By modulating the mid-IR perturbation signals, the SERS signal at the visible wavelength emitted from the molecules within the gap between the Au sphere and the planar film can be controlled, which is similar to the general principle of optical modulators and/or optical switches. Relying on advanced characterization capabilities (i.e., the single-photon lock-in detection scheme), this work observed the time-dependent rise and decay of the SERS signal in a few hundred nano-seconds, opening a way to explore the optical manipulation of SERS signals of trace chemical molecules using mid-IR perturbation.

**Fig. 1:**
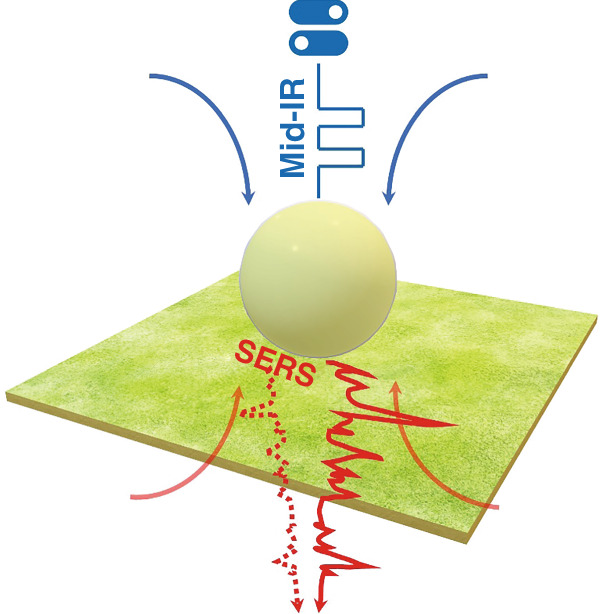
Plasmonic nanocavity for mid-IR perturbation. Schematic illustration to trigger the SERS signal from a nanocavity using a mid-IR perturbation^15^

Although the repeatability of this sophisticated characterization needs further improvement, this combined sensing system with nanophotonic structures and advanced detection schemes indicates a new regime for fundamental investigation. Remarkably, it opens the door to manipulate the optical signature of chemical molecules from tiny volumes and study new mechanisms within quantum regimes. It will also enable the exploration of building blocks for efficient light trapping and localized field concentration that will yield important technological breakthroughs for on-chip integrated photonics and optical modulators and sensors in bio/chemical systems.
